# Rapid Quantitative Detection of Deltamethrin in *Corydalis yanhusuo* by SERS Coupled with Multi-Walled Carbon Nanotubes

**DOI:** 10.3390/molecules25184081

**Published:** 2020-09-07

**Authors:** Hui Zhang, Pengcheng Nie, Zhengyan Xia, Xuping Feng, Xiaoxi Liu, Yong He

**Affiliations:** 1 College of Biosystems Engineering and Food Science, Zhejiang University, Hangzhou 310058, China; 21813051@zju.edu.cn (H.Z.); npc2012@zju.edu.cn (P.N.); pimmmx@163.com (X.F.); 21813015@zju.edu.cn (X.L.); zjuheyong@sina.com (Y.H.); 2 Key Laboratory of Spectroscopy Sensing, Ministry of Agriculture, Hangzhou 310058, China; 3 West Electronic Business Company Limited, Yinchuan 750000, China; 4 School of Medcine, Zhejiang University City College, Hangzhou 310015, China

**Keywords:** Chinese herbal medicines, pesticide residues, surface-enhanced Raman spectroscopy, pretreatment method, prediction models

## Abstract

With the increase in demand, artificially planting Chinese medicinal materials (CHMs) has also increased, and the ensuing pesticide residue problems have attracted more and more attention. An optimized quick, easy, cheap, effective, rugged and safe (QuEChERS) method with multi-walled carbon nanotubes as dispersive solid-phase extraction sorbents coupled with surface-enhanced Raman spectroscopy (SERS) was first proposed for the detection of deltamethrin in complex matrix *Corydalis yanhusuo*. Our results demonstrate that using the optimized QuEChERS method could effectively extract the analyte and reduce background interference from *Corydalis*. Facile synthesized gold nanoparticles with a large diameter of 75 nm had a strong SERS enhancement for deltamethrin determination. The best prediction model was established with partial least squares regression of the SERS spectra ranges of 545~573 cm^−1^ and 987~1011 cm^−1^ with a coefficient of determination (*R*^2^) of 0.9306, a detection limit of 0.484 mg/L and a residual predictive deviation of 3.046. In summary, this article provides a new rapid and effective method for the detection of pesticide residues in CHMs.

## 1. Introduction

Chinese herbal medicines (CHMs) have played an important role in clinical therapy for many diseases, especially as a valuable, readily available resource for healthcare for thousands of years. For example, the alkaloids in *Corydalis* (*Corydalis yanhusuo* W.T. Wang) are one of the commonly used CHMs in traditional medical treatment as well as modern Western medical treatment for cancer, asthma, malaria, and other diseases [1,2]. However, the commercial cultivation of CHMs leads to the frequent application of diverse pesticides to prevent, repel or mitigate the effects of pests, which causes environmental pollution and threatens human health [3,4]. With the wide application of corydalis in the medical field, it is crucial to examine pesticide residues for safety. Deltamethrin is a widely used pyrethroid insecticide, which has a good insecticidal effect on weevils and other insects that occur during the growth of *Corydalis*. Most of the pesticides applied on farmland will diffuse into the soil, and some will be adsorbed into *Corydalis* [5]. Therefore, the detection of deltamethrin in *Corydalis* is carried out.

Currently, the detection methods for pesticide residues in CHMs are mainly chromatography [6,7] and chromatography-mass spectrometry [8,9]. Yang et al. (2013) used capillary gas chromatography to determine 19 organochlorine pesticide residues in *Corydalis*, and the results confirmed that *Corydalis* contained different levels of pesticide residues [10]. Liu et al. (2016) compared two extraction methods for the determination of 135 pesticides in *Corydalis* Rhizoma, *Chuanxiong* Rhizoma and *Angelicae Sinensis* Radix by liquid chromatography-triple quadrupole mass spectrometry [11]. Zhao et al. (2017) used ultra-high-performance liquid chromatography-tandem mass spectrometry to detect 19 pesticide residues in *Corydalis* coupled with acetonitrile extraction and multi-walled carbon nanotube purification [12]. In these studies, the methods have the advantages of high sensitivity, good repeatability, and accuracy for multi-residue analysis in CHMs. However, these methods rely on expensive large-scale instruments. Moreover, the detection process is always a high-cost, long and cumbersome operation that makes it difficult to detect pesticide residues quickly for real-time application.

Surface-enhanced Raman spectroscopy (SERS) is a means of enhancement for Raman spectroscopy, which can reflect the structural information inside the molecule and has a near single-molecule detection sensitivity and fingerprinting capability [13,14]. SERS is a new method for pesticide residue detection that is considered to be sensitive, effective, anti-interference and non-destructive [15,16]. In addition, advances in portable spectrometers and substrates have made real-time and on-site detection and analysis of pesticide residues a reality [17]. This method is also used more and more for food and environmental safety detection [18,19]. Huang et al. (2015) combined SERS technology with a rapid pretreatment quick, easy, cheap, effective, rugged and safe (QuEChERS) method to detect chlorpyrifos pesticide residues in rice, and the limit of detection of chlorpyrifos in rice was less than 0.506 mg L^−1^ [20]. Wang et al. (2017) developed a flexible and absorbent wiping SERS substrate, which is used to quickly detect thiram residues in apples, pears and grapes [21]. Zhai et al. (2017) used SERS for the determination of acetamiprid, chlorpyrifos and carbendazim mixed pesticides in apple samples, which provides an ultra-sensitive SERS performance for the simultaneous quantification of multiple residual pesticides in apple samples [22]. Dong et al. (2018) applied SERS based on a gold nanoparticle (AuNP) substrate to detect deltamethrin in strawberries with a detection limit that reached 0.1 mg L^−1^ [23]. However, the composition of CHMs are complex and contain a wide variety of biologically active compounds, including sugars, peptides, sterols, labels and alkaloids, which bring great background interference to the detection of pesticide residues [24]. To our knowledge, no report has explored the possible application of SERS for residual pesticides in CHMs.

The purpose of this study was to optimize the use of a modified quick, easy, cheap, effective, rugged and safe (QuEChERS) method followed by SERS to detect deltamethrin in the CHMs *Corydalis*. In this paper, the characteristic peaks of deltamethrin were assigned. The complex matrix of *Corydalis* was pretreated and then detected by SERS with high-sensitivity stable gold nanoparticles (AuNPs) as the substrate. SERS spectral data were acquired and quantitative models were established using statistical tools including partial least squares regression (PLSR).

## 2. Results and Discussion

### 2.1. Raman Spectra of Deltamethrin

**Figure 1:**
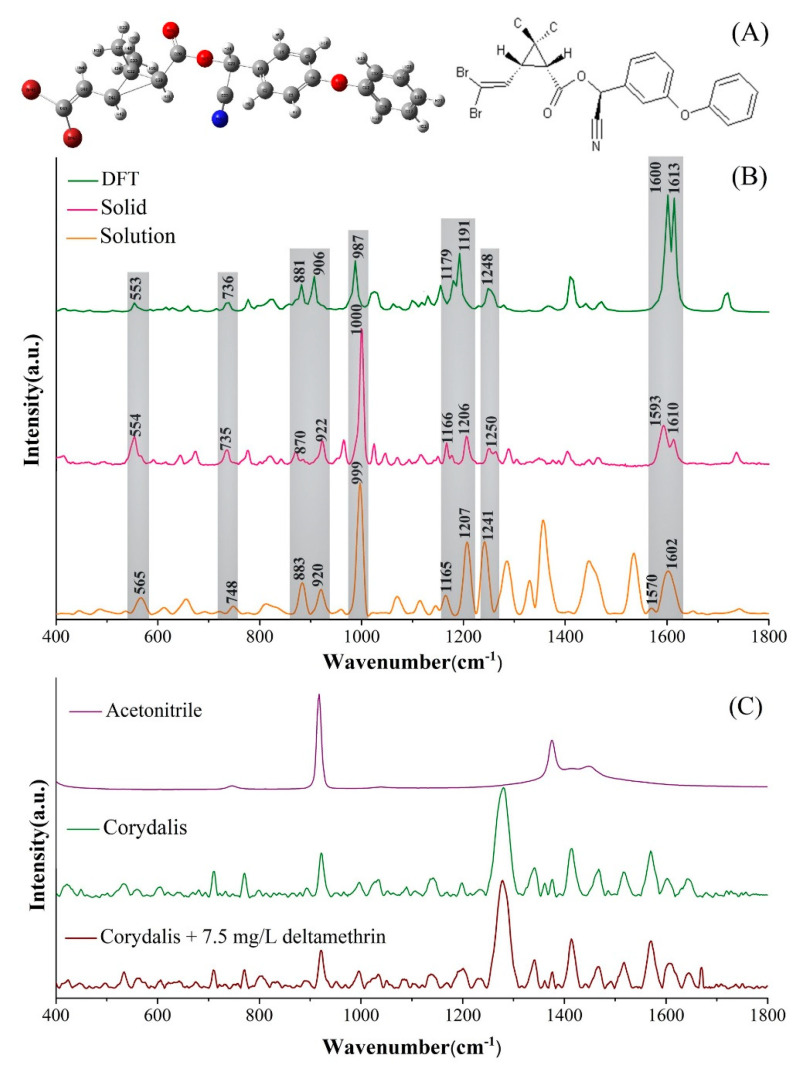
Raman peak assignment of deltamethrin. (**A**) molecular structure of deltamethrin; (**B**) Comparison of Raman spectrum simulated by density functional theory (DFT), Raman spectrum of deltamethrin solid and surface-enhanced Raman spectroscopy (SERS) spectrum of 10 mg/L deltamethrin acetonitrile solution; (**C**) SERS spectra of acetonitrile, extract of no-treatment *Corydalis* and extract of 7.5 mg/L deltamethrin-containing *Corydalis*.

The molecular structure of deltamethrin, calculated by density functional theory (DFT), is shown in Figure 1A. It can be seen that the molecular structure of deltamethrin mainly consists of C-Br, C=C, C=N, C=O, C-H, N-H and a benzene ring. The theoretical Raman spectra of deltamethrin, calculated by DFT, the Raman spectra of standard deltamethrin solid and SERS of 10 mg/L deltamethrin solution are shown in Figure 1B. It is clear that the spectra obtained from the experiment have a high matching degree with the theoretical Raman spectrum at the peak position in the range of 400~1800 cm^−1^. Although there were different degrees of Raman shift between them, the peak position and peak intensity of deltamethrin have a good corresponding relationship, especially between the DFT simulation and the standard solid deltamethrin. According to the DFT-simulated results, the assignments of the Raman spectra of deltamethrin are listed in Table S1.

The characteristic peaks of deltamethrin in SERS were mainly 565, 748, 883, 920, 999, 1165, 1207, 1241 and 1602 cm^−1^ (Figure 1B and Table S1). According to the DFT simulation results, the peak at 999 cm^−1^ was assigned to the stretching vibration of the benzene ring and the deformation vibration of C-C. The peak at 559 cm^−1^ was assigned to C-Br and C-C stretching vibrations. The peaks at 748 cm^−1^ and 883 cm^−1^ were assigned to a C-H deformable vibration. The peaks at 920 cm^−1^, 1165 cm^−1^ and 1241 cm^−1^ were assigned to a C-H stretching and deformable vibration. The peak at 1207 cm^−1^ was assigned to a C-C stretching vibration. The peak at 1602 cm^−1^ was assigned to a C=C stretching vibration. The attribution of these peaks is highly consistent with the attribution results in other research, and can be used as the SERS characteristic peaks of deltamethrin [25,26].

Furthermore, we evaluated the Raman spectra characteristics of deltamethrin residue in *Corydalis*. The spectra of the deltamethrin and *Corydalis* extract obtained by the Raman spectrometer are shown in Figure S1. Except for the 2252 cm^−1^ assigned to the solvent acetonitrile, the Raman peak is mainly in the 400–1700 cm^−1^ band. Therefore, this article analyzes the Raman spectra in the 400–1700 cm^−1^ band. No-treatment *Corydalis* and the *Corydalis* containing 7.5 mg/L deltamethrin were separately extracted with acetonitrile. Figure 1C shows the SERS of the extract of deltamethrin-containing *Corydalis*, the SERS of the extract of no-treatment *Corydalis* and the Raman spectrum of acetonitrile. Compared to the SERS spectrum of deltamethrin standard solution, the deltamethrin testing solution extracted from *Corydalis* contained more Raman peaks. The matrix of *Corydalis* contains a variety of substances, including more than a dozen alkaloids, which can cause serious interference in detection [27]. Through a literature comparison, the assignments of the Raman spectra of *Corydalis* extract are listed in Table S2 [28,29,30,31,32,33,34,35,36]. However, the presence of these substances could affect the detection of Raman spectroscopy, even if the peaks of *Corydalis* extract directly covered the peaks of deltamethrin. Therefore, pretreatment technique is required to remove excess substances to eliminate serious background interference.

**Figure 2:**
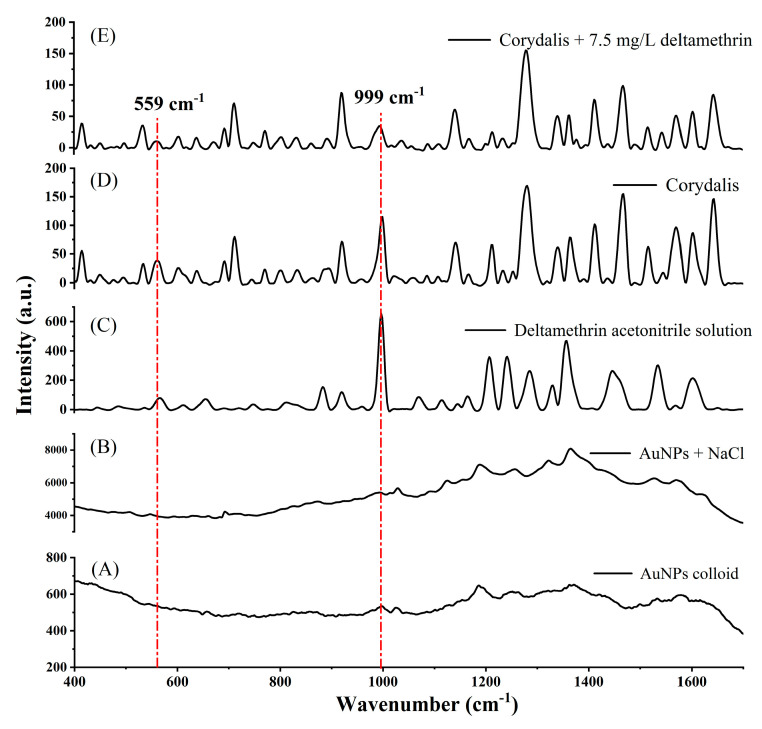
Selection of Raman characteristic peaks for detection of deltamethrin in *Corydalis*. (**A**) Raman spectrum of AuNPs colloid; (**B**) Raman spectrum of AuNPs with NaCl; (**C**) SERS of deltamethrin acetonitrile solution; (**D**) SERS of *Corydalis* extract; (**E**) SERS of *Corydalis* extract with 7.5 mg/L deltamethrin.

In order to detect deltamethrin in *Corydalis*, it is necessary to find characteristic peaks that can be used for detection. The extract was treated with anhydrous magnesium sulfate (MgSO_4_), primary secondary amine (PSA), C_18_ and graphitized carbon black (GCB) as a dispersed solid-phase sorbent, and the results are shown in Figure 2. The characteristic peak needs to meet the following criteria: not affected by colloid (Figure 2A), aggregation agent (Figure 2B) and solvent (acetonitrile); corresponding to the peak of the deltamethrin standard solution (Figure 2C); the peak intensity of the extract containing deltamethrin (Figure 2D) needs to be stronger than the extract without deltamethrin (Figure 2E). By comparison, the peak at 999 cm^−1^ can be used as the main characteristic peak for deltamethrin detection in *Corydalis*, and the peak at 559 cm^−1^ can be used as the secondary characteristic peak.

### 2.2. SERS Enhanced Substrate

**Figure 3:**
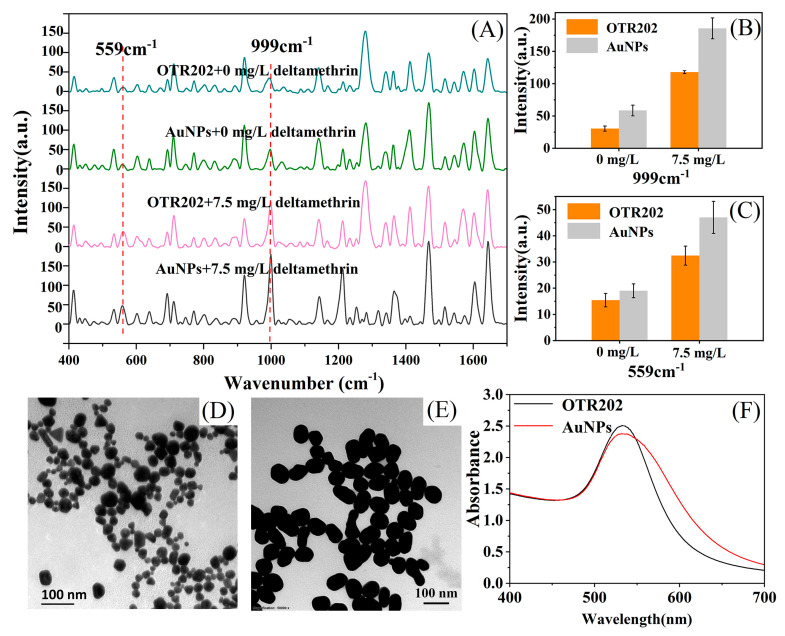
Surface-enhanced Raman spectroscopy (SERS) substrate characterization and comparison. (**A**): SERS of purified extracts using Opto Trace Raman 202 (OTR202) and gold nanoparticles (AuNPs) as substrates; (**B**,**C**): peak intensity of two substrates at 999 cm^−1^ and 559 cm^−1^, respectively; (**D**,**E**): TEM of OTR202 and synthetic AuNPs, respectively; (**F**): UV-vis absorption spectrum of two substrates.

In this experiment, the commercial Opto Trace Raman 202 (OTR202) substrate and the synthetic AuNP colloid substrate were used for detection, and the aggregation agents were (Opto Trace Raman 202) OTR103 and 1% NaCl solution, respectively. OTR202 has been proven to be an effective SERS active substrate, which can be used to detect and analyze a variety of substances [20,37]. From Figure 3A–C, it was found that the enhancement effect of AuNPs at peaks of 559 cm^−1^ and 999 cm^−1^ was significantly better than that of OTR202. Figure 3D,E are transmission electron micrographs of OTR202 and AuNPs, respectively. Figure S2 shows the statistics of the two substrate particle sizes, and the results show that the particles in the self-made AuNP colloid were more uniform and the particles were larger. The average size of the self-made gold nanoparticles was 75 nm, while the OTR202 was only 30 nm. Comparing the UV-Vis absorption spectra of OTR202 and AuNP colloids in Figure 3F, it can be found that the absorption peak of the self-synthesized AuNPs colloid was red-shifted, which could also demonstrate that the diameter of AuNPs was larger [38,39,40].

As shown in Figure S3, the Raman spectrum of 2 × 10^−2^ M deltamethrin only had a faint signal. However, the deltamethrin at 999 cm^−1^ with a concentration of 2 × 10^−7^ M is still obvious using 75 nm AuNPs as SERS substrates. In this work, the enhancement factor (EF) of deltamethrin at 999 cm^−1^ was calculated from Equation (1) [41,42,43]:

(1) $$\mathrm{EF}=\frac{I_{\mathrm{SERS}}\times C_{\mathrm{Raman}}}{I_{\mathrm{Raman}}\times C_{\mathrm{SERS}}}$$

where $I_{\mathrm{SERS}}$ is the integrated intensity of deltamethrin molecules adsorbed on the substrate surface. $I_{\mathrm{Roman}}$ is the integrated intensity of the same Raman band obtained without the AuNPs. $C_{\mathrm{SERS}}$ represents the concentration of deltamethrin adsorbed on SERS AuNPs. $C_{\mathrm{Raman}}$ is the concentration of deltamethrin with an acceptable normal Raman spectroscopy. The EF of deltamethrin at 999 cm^−1^ is 1.8 × 10^5^. Compared with the research results of Qin et al. [44] and Sukmanee et al. [45], the enhancement effect increased.

The SERS substrate is one of the key factors that determine the success of the detection. At present, the research of SERS substrates focusses on improving the sensitivity, reproducibility, stability, uniformity and flexibility. Great progress has been made on SERS substrates, including various types of materials and nanostructures, as well as flexible and convenient 2D and 3D substrates [46,47,48,49,50]. The AuNP colloid is the most stable and controllable SERS substrate, and the larger and more uniform the particle size, the better and more stable the enhancement effect [51,52]. The sample molecules can condense the sol, resulting in the Surface Plasmon Resonance effect, which greatly enhances the electromagnetic field [53]. Another aspect, the interaction and synergistic effect between the metal nanoparticles, will further enhance the local electromagnetic field around the metal nanoparticles, forming a “hot spot”, which also enhances the SERS signal [54]. When colloids are used directly, appropriate inorganic salts are commonly used as aggregation agents to generate sufficient NP junctions for SERS detection [55].

### 2.3. Pretreatment Method Optimization

#### 2.3.1. Optimization of Extraction Conditions

**Figure 4:**
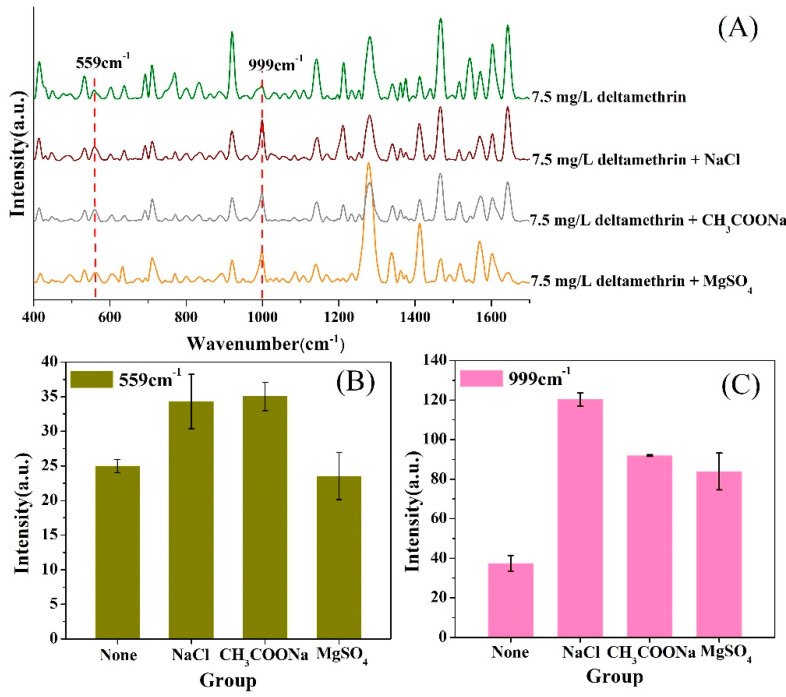
Comparison of water removal methods. (**A**) SERS spectra characters after removing water method by adding with sodium chloride, anhydrous sodium acetate and anhydrous magnesium sulfate. (**B**) peak intensity of 559 cm^−1^ under different water removal modes; (**C**) peak intensity of 999 cm^−1^ under different water removal modes.

In this research, we compared the water removal methods by adding sodium chloride, anhydrous sodium acetate and anhydrous magnesium sulfate. Figure 4A shows the SERS spectra characteristics effected by different removal methods. The intensity variation in the two characteristic peaks (559 and 999 cm^−1^) can be seen in Figure 4B,C. The results show that the effect of sodium chloride is obviously better than the others. In this experiment, after adding anhydrous magnesium sulfate to the sample, agglomeration and exothermic phenomena occurred, which resulted in a decrease in the effect of removing water and the decomposition of the pesticide having poor heat resistance. Anhydrous sodium acetate obviously deepened the color of the extract, indicating that more substances were precipitated, resulting in serious background interference, and thus the intensity of the characteristic peak was weakened. Therefore, sodium chloride was finally selected to stratify the aqueous phase and the organic phase, avoiding agglomeration and heat release without changing the pH of the extract; therefore, the extraction process was more stable.

#### 2.3.2. Optimization of Purification Conditions

**Figure 5:**
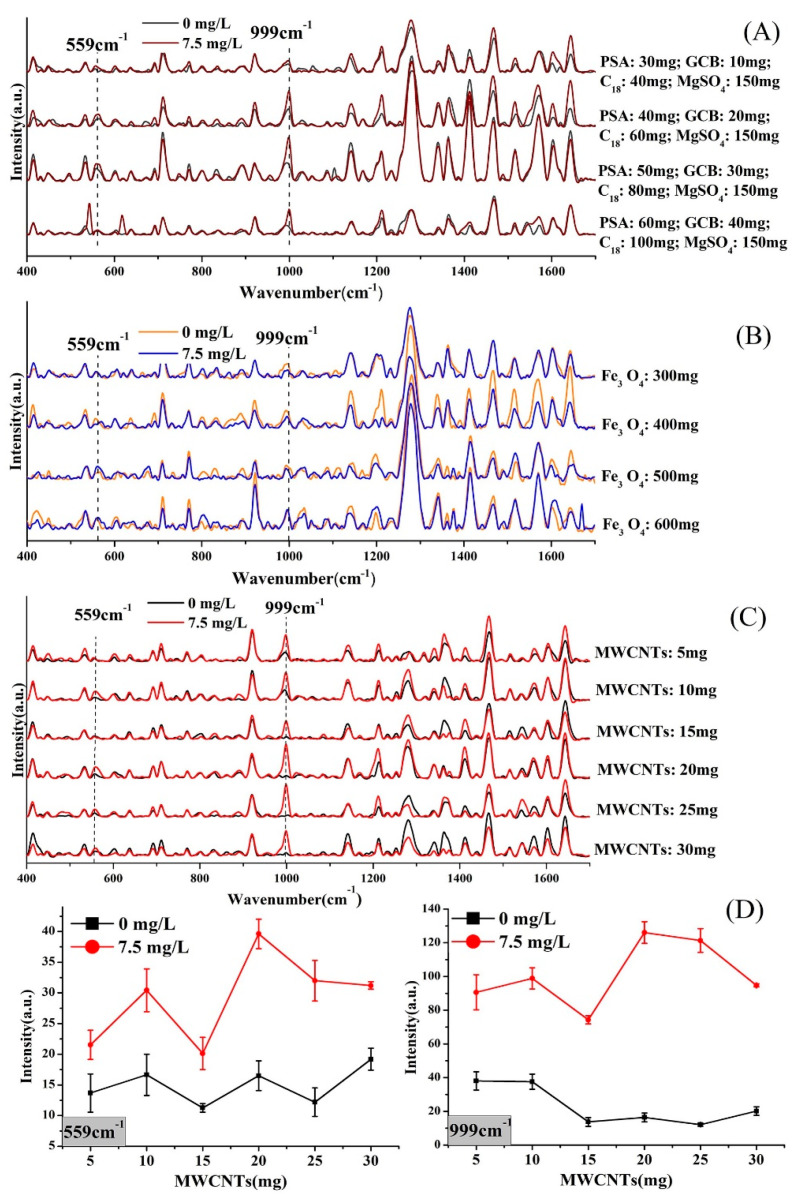
The choice of purification conditions. (**A**) SERS of extracts purified by primary secondary amine (PSA), C_18_, graphitized carbon black (GCB) and anhydrous MgSO_4_; (**B**) SERS of extracts purified by Fe_3_O_4_ with a particle size of 100 nm; (**C**) SERS of extracts purified by multi-walled carbon nanotubes (MWCNTs); (**D**) peak intensity after purification by different amount of MWCNTs at 999 cm^−1^ and 559 cm^−1^.

Figure 5 shows the SERS spectra of the 1-mL extracts of *Corydalis* samples purified by different methods. The materials MgSO_4_, PSA, C_18_ and GCB are not suitable for purifying the extract of *Corydalis* (Figure 5A). The peak intensity at 559 cm^−1^ and 999 cm^−1^ was still strong without the addition of deltamethrin (Figure S4). Additionally, there is no obvious characteristic of deltamethrin in the SERS spectral by adjusting the amount of Fe_3_O_4_ (Figure 5B), indicating that Fe_3_O_4_ with a particle size of 100 nm does not have a purifying effect on the extract of *Corydalis*. MgSO_4_, PSA, C_18_ and GCB are the most commonly used purification materials for QuECHERS [56,57]. Anhydrous MgSO_4_ has a good ability to remove water. C_18_ has a hydrophobic effect and can adsorb non-polar components, and PSA can remove organic acids, pigments, and phenolic compounds, and it has no effect on detection [58]. Although PSA has the ability to remove pigments, the removal is not ideal. GCB has a strong adsorption capacity for pigments, but it also has an adsorption effect on pesticides, which leads to a decrease in pesticide recovery. At present, the combination of these four materials has a good purification effect on the detection of pesticides in fruits and vegetables. In the experiment, it was found that the yellow color of the extract was slightly lighter after being purified, but it still had a deep yellow color. This indicates that PSA and GCB rarely adsorb alkaloid pigments. In addition, most alkaloids are polar compounds, so C_18_ does not adsorb them. In short, the CHM matrix is more complex than fruits and vegetables, and conventional purification materials cannot meet the requirements. In recent years, Fe_3_O_4_, as one of the magnetic nanoparticles, has been used in the pretreatment process for pesticide residues due to its unique properties such as nanometer-scale particle size, unique magnetic properties, and quantum size effects [59,60]. However, according to the experimental results, Fe_3_O_4_ did not adsorb the extract of *Corydalis* containing a variety of alkaloids.

Figure 5C shows the SERS spectral of the 1 mL extracts of *Corydalis* samples purified by multi-walled carbon nanotubes (MWCNTs). The characteristic peak intensity of the sample containing deltamethrin was much stronger than that of the no-treatment sample, while the characteristic peak intensity of the no-treatment sample was weak. The results indicate that some of the interfering substances were effectively removed by MWCNTs. Figure 5D shows the peak intensity after purification by different amounts of MWCNTs at 999 cm^−1^ and 559 cm^−1^. According to Figure 5D, when the amount of MWCNTs was 20 mg, the purification achieved the best results with the strongest intensity found in two peaks of the deltamethrin-containing sample and the weakest intensity found in two peaks of the no-treatment sample. When the dosage was less than 20 mg, the purification was not complete, while, when it was more than 20 mg, the adsorption of deltamethrin increased, and the characteristic peak intensity was also reduced. Finally, by experimental comparison, the amount of MWCNTs used to purify 1 mL of the extract was determined to be 20 mg. MWCNTs have been increasingly used for pretreatment in recent years. MWCNTs have the characteristics of a nano-scale hollow tubular structure, large specific surface area, strong adsorption capacity, stability and durability. MWCNTs have a nanometer-scale hollow tubular structure and a large specific surface area, so they have a strong adsorption capacity. They have become one of the enrichment and purification materials for detecting pesticide residues [61,62]. Alkaloids are nitrogen-containing organic compounds, most of which have complex ring structures. The carbon atoms in MWCNTs form π bonds, and their π electrons can combine with compounds containing π electron structures (such as benzene rings) through π–π non-covalent bonding. Thus, MWCNTs have a good adsorption effect on alkaloids. This experiment also shows that MWCNTs have a good adsorption effect on active substances such as alkaloids in complex matrix CHMs. However, they also have an adsorption effect on pesticides. Therefore, when using MWCNTs as purification materials, attention should be paid to their dosage.

### 2.4. Detection of Deltamethrin in Corydalis

**Figure 6:**
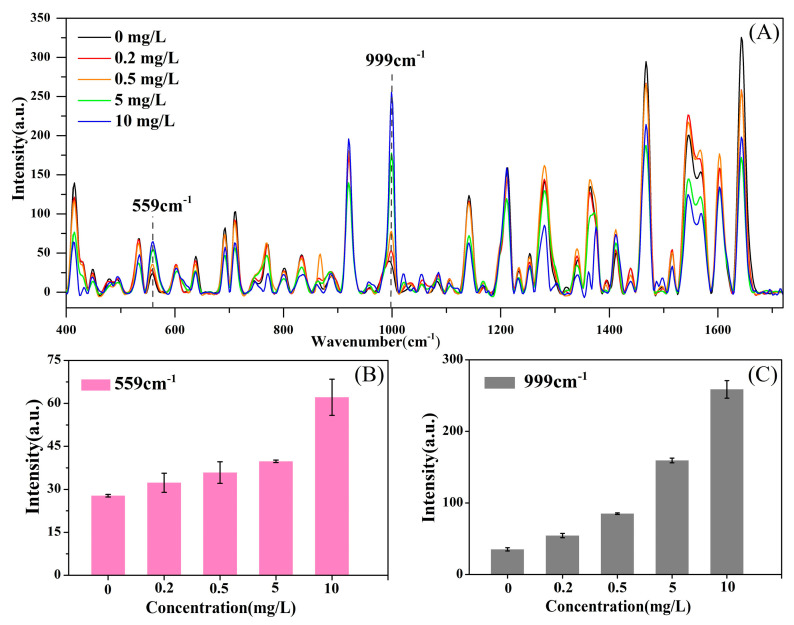
Raman spectra of different levels of deltamethrin added to *Corydalis*. (**A**) SERS of deltamethrin concentration adding in *Corydalis* sample from 0 to 10 mg/L; (**B**) peak intensity of 559 cm^−1^ under different levels of deltamethrin; (**C**) peak intensity of 999 cm^−1^ under different levels of deltamethrin.

Figure 6A shows the SERS characteristics with an increase in the deltamethrin concentration added to the *Corydalis* sample from 0 to 10 mg/L. The intensity of two characteristic peaks, 559 cm^−1^ and 999 cm^−1^, were increased with the increasing concentration of deltamethrin (Figure 6B,C).

To analyze the mathematic relationship between Raman spectral data and deltamethrin concentration, linear regression equations were calculated on the characteristic peak intensity and deltamethrin concentration, and PLSR models were established based on characteristic peak curve data. The actual values of 75 samples were obtained using HPLC. The data range is 0.143–9.773 mg/L. The method recovery rate was mainly 60–100%. The deltamethrin concentrations used in the modeling analysis were the actual values. In the PLSR model, the ratio of prediction set to calibration set is 1:2.

**Figure 7:**
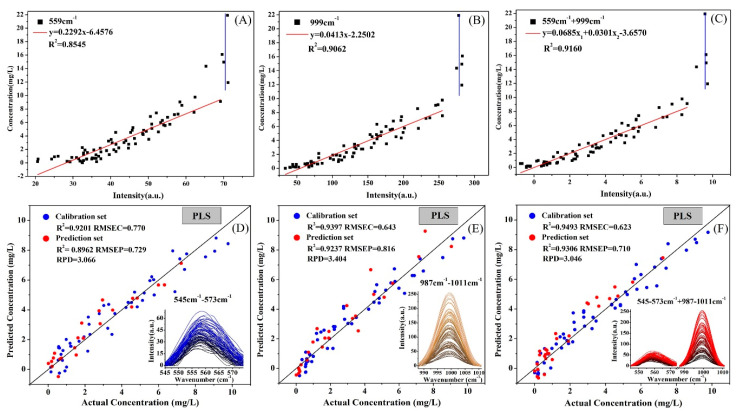
Quantitative detection models of deltamethrin in *Corydalis*. Linear regression equation between peak intensity and deltamethrin content in *Corydalis*: (**A**) 559 cm^−1^; (**B**) 999 cm^−1^; (**C**) 559 cm^−1^ + 9 99 cm^−1^. Partial least squares regression (PLSR) model based on spectral data of characteristic peak bands and deltamethrin content: (**D**) 545~573 cm^−1^; (**E**) 987~1011 cm^−1^; (**F**) (545~573 cm^−1^) + (987~1011 cm^−1^).

Figure 7A–C illustrates the linear regression equations established for the peak intensities of 559 cm^−1^, 999 cm^−1^, and 559 + 999 cm^−1^ and the deltamethrin content, respectively. The results show that the peak intensity had a good linear relationship with the deltamethrin concentration in the range of 0–10 mg/L. The *R*^2^ between the intensity of the characteristic peaks and the deltamethrin concentration in the range of 0–10 mg/L were all above 0.8545, and the correlation at 999 cm^−1^ is better than 559 cm^−1^. The correlation between the combination of the two peaks and the deltamethrin was the best, and the *R*^2^ reached 0.9160. However, when the concentration was above 10 mg/L, the peak intensity reached saturation and tended to be gentle, and no longer increased with the concentration.

Figure 7D–F shows the PLSR models for spectral data of 545~573 cm^−1^ (559 cm^−1^ ± 14 cm^−1^) near 559 cm^−1^ and the spectral data of 987~1011 cm^−1^ (999 cm^−1^ ± 12 cm^−1^) near 999 cm^−1^ and deltamethrin content, respectively. The results show that the best predictive effect in the deltamethrin quantitative analysis model was the PLS model based on the spectral data of a combination of the two peak bands, and the correlation coefficient of determination (*R*^2^), root mean square error for prediction (RMSEP) and residual predictive deviation (RPD) were 0.9306, 0.710 and 3.046, respectively. In addition, the prediction effect of the model at 999 cm^−1^ was better than 559 cm^−1^, which was consistent with the linear relationship between peak intensity and deltamethrin concentration.

The detection limit of the PLS models was determined by the equation based on the standard error in the y-intercept and slope [63]. The detection limit results are shown in Table S3. The detection limits calculated based on the three PLS models were all below 0.668 mg/L. The optimal was also the detection limit of the PLS model based on the combination of the two peak bands, and the detection limit reached 0.484 mg/L. However, the calculated results are higher than the detection limits obtained by direct observation (Figure 6), which may be due to the large fluctuations in peak intensity at low concentrations. Therefore, the detection limit of the low concentration (0–1 mg/L) was analyzed based on three times the standard deviation of the blank sample (Figure S5). The detection limit of deltamethrin at 999 cm^−1^ reached 0.186 mg/L, while at 559 cm^−1^ it reached 0.617 mg/L. The results show that the calculation results based on the PLSR model mentioned above are reliable.

To the best of our knowledge, up to now, there have been no applications of SERS or other quick detection methods to determine the pesticide residues in CHMs. Compared with traditional methods (gas chromatography, gas chromatography-mass spectrometry, liquid chromatography, liquid chromatography-mass spectrometry), this method can shorten the detection time (including sample pretreatment) to less than half an hour, and can realize batch detection. Moreover, using MWCNTs instead of solid-phase extraction columns, as well as adsorbents such as PSA, C_18_, and GCB, can reduce costs by dozens of times. However, the model accuracy and detection sensitivity are worse than traditional methods. At present, there is still a lack of standards for the minimum residues of pesticides in CHMs, but they are usually lower than 0.5 mg/kg. This method can essentially meet the demand, but the recovery rate, stability and detection sensitivity of the method still need to be explored and optimized further.

## 3. Materials and Methods

### 3.1. Chemicals and Reagents

In this experiment, dried *Corydalis* samples were purchased from CHM growers of Zhejiang province, China. The deltamethrin (C_22_H_19_Br_2_NO_3_, 99.6% purity) was supplied by Sigma-Aldrich (Beijing, China). The acetonitrile (C_2_H_3_N, chromatographically purity) was from Amethyst Chemicals (Beijing, China). Chloroauric acid (HAuCl_4_, 99.999% purity), trisodium citrate (Na_3_C_6_H_5_O_7_, Analytical Pure), anhydrous magnesium sulfate (MgSO_4_, Analytical Pure), anhydrous sodium acetate (CH_3_COONa, Analytical Pure) and sodium chloride (NaCl, Analytical Pure) were purchased from National Standards Information Center (Beijing, China). PSA, C_18_, graphitized GCB, Fe_3_O_4_ nanoparticles and multi-walled carbon nanotubes (MWCNTs) were purchased from Agela Technologies (Beijing, China). Water was collected in a laboratory grade ultrapure water instrument (EPED Technology Co., Ltd., Nanjing, China). The OTR 202 and OTR 103 was supplied by Opto Trace Technologies, Inc. (Suzhou, China).

### 3.2. Preparation of the Sample

Different level solutions of 200, 100, 50, 10 and 5 mg/L of deltamethrin were prepared with acetonitrile as the solvent. Different amounts of the deltamethrin solution were added to an accurately weighed 2 g of *Corydalis*, and dried with nitrogen. Then, the QuCHERS method was used to pretreat the samples. For the extraction procedure, each 2 g *Corydalis* sample was first mixed with 2 mL pure water in 10 mL centrifuge tube and vortexed for 30 s. Thereafter, 4 mL acetonitrile was added into the centrifuge tube, followed by vortexing for 30 s and then sonicated for 10 min. After that, water removal material was added to different samples in order to remove water. Finally, the solution was vortexed for 1 min and then centrifuged for 3 min, and the supernatant was taken for purification. For the purification procedure, 1 mL of the extract was, respectively, placed in a 2 mL centrifuge tube containing purification materials, followed by vortexing for 1 min, and centrifuging at 10,000 r/min for 5 min. The supernatant was collected and placed at 4 °C for SERS measurement.

We optimized the extraction and purification steps separately to achieve the best experimental results. During the extraction process, the experiment compared the water removal methods of sodium chloride (2 g), anhydrous sodium acetate (2 g) and anhydrous magnesium sulfate (2 g). Meanwhile, we proposed three purification methods. The first was different amounts of magnesium sulfate (150 mg), PSA (30, 40, 50 and 60 mg), C_18_ (40, 60, 80 and 100 mg) and GCB (10, 20, 30 and 40 mg). The second was Fe3O4 with a particle size of 100 nm (300, 400, 500 and 600 mg). The last one was different amounts of MWCNTs (5, 10, 15, 20, 25 and 30 mg).

### 3.3. Preparation of AuNPs

In this study, the synthesis of AuNPs was achieved by a chemical reduction method [64]. We quickly added 0.7 mL of 1% Na_3_C_6_H_5_O_7_ into 100 mL boiling HAuCl_4_ at a concentration of 0.01% in a intelligent thermostat magnetic stirrer (Zhengzhou Ya-Rong Instrument Co., Ltd., Zhengzhou, China). The solution was then heated and stirred continuously for 20 min at the boiling state. Finally, the prepared gold colloid was placed in a brown jar and stored in the dark at room temperature.

Optical absorption measurements of AuNPs were carried out on a TU-1010 ultraviolet spectrophotometer (Beijing General Instrument Co., Ltd., Beijing, China). The morphological features of the prepared AuNPs structures were characterized with the FEI Tecnai G2 F20 S-TWIN transmission electron microscope (TEM, USA FEI Corporation, Hillsboro, OR, USA).

### 3.4. Raman Spectrum Acquisition

The RmTracer-200-HS portable Raman spectrometer equipped with a 785-nm laser (Opto Trace Technologies, Inc., Suzhou, China) was used to obtain the Raman spectra. The acquisition time was 10 s with 3 accumulations. The laser power used was 100 mW. The scanning range was 300 to 3200 cm^−1^ and the optical resolution was 2 cm^−1^. Standard deltamethrin powder was placed on a quartz plate with glass slides flattened to collect the Raman spectra. Five hundred µL of the colloid substrate, 100 µL of the test solution, and 100 µL of the 1% NaCl solution were added to a 2 mL quartz bottle, followed by vortexing for 5 s, then placed in a liquid sample pool to collect the SERS of the sample.

### 3.5. HPLC Measurement

The content of deltamethrin in the sample were determined by HPLC as a true value. The chromatographic column used was a Zorbax Eclipse XDB C_18_ column (2.1 × 150 mm, 5 μm, Santa Clara, CA, USA). The elution system consisted of 0.1% formic acid solution (A) and methanol (B). The gradient was 0–11 min, 1% A–75% A. The detection wavelength was 300 nm. The flow rate was 0.3 mL/min. The injection volume was 10 uL and the column temperature was 35 °C.

### 3.6. Data Analysis

Density functional theory (DFT) is a quantum mechanical method for studying the electronic structure of multi-electron systems. DFT is one of the most commonly used methods in the field of condensed matter physics and computer chemistry [65]. It can be used to study the properties of molecules and is currently widely used in spectroscopy [18,23]. In this paper, the Becke three-parameter Lee-Yang-Parr (B3LYP) functional with the 6-31G (d,p) basis set was used to simulate deltamethrin pesticide molecules.

We used the software Ominic (Thermo Fisher Scientific Inc., Waltham, MA, USA) to preprocess the SERS spectra, including smoothing and baseline calibration. Then, PLSR was applied to construct a deltamethrin prediction model based on Raman spectral data. PLSR is one of the most widely used regression modeling methods in spectral data analysis since it can efficiently and reliably process complex spectral data [23]. PLSR linearly transforms raw data into mutually orthogonal and unrelated latent variables, also known as principal components. The first few latent variables of PLS contain the main information of the original data and can explain most of the variables. The correlation coefficient of determination (*R*^2^), root mean square error for prediction (RMSEP) and residual predictive deviation (RPD) are the main parameters for evaluating the prediction models. A higher *R*^2^ value and lower RMSEP value represent that the results from the prediction model are more reliable. The quality of the PLSR model can be evaluated by the RPD values. RPD values of 5–10 are satisfactory for quality control purposes, while values of 2.5–5 are sufficient for screening samples [66].

## 4. Conclusions

In this paper, the SERS technology with facile synthesized AuNPs as the substrate coupled with the optimized QuECHERS pretreatment method with MWCNTs as the purification material realized the quantitative detection of deltamethrin in CHMs *Corydalis*. The characteristic peaks of deltamethrin were assigned by the simulation of deltamethrin molecules by DFT. The complex matrix of CHMs causes severe background interference, making it is difficult for SERS to detect. By optimizing the extraction and purification conditions, we decided to extract with acetonitrile and sodium chloride, and purify with MWCNTs. The synthesized AuNPs with a large diameter of 75 nm have a good uniformity and SERS enhancement effect. Finally, based on the characteristic peak intensities at 559 and 999 cm^−1^ and characteristic peak spectral band, the quantitative analytical equations of deltamethrin in *Corydalis* were established. The model obtained by combining the two characteristic peaks showed a good prediction effect. The calculated detection limit was 0.484 mg/L. In summary, optimized QuEChERS with the SERS method can enable the rapid and reliable quantitative/qualitative analysis of the target analytes in CHMs. Our future work will focus on applying SERS to CHMs for further pesticide residue detection.

## Supplementary Materials

The Supplementary Materials can be found online: Figure S1: SERS spectra of deltamethrin solution and corydalis extract. Figure S2. Size distribution of nanoparticles. Figure S3: Raman spectroscopy of deltamethrin. Figure S4: Peak intensity at 999 cm^−1^ and 559 cm^−1^ with MgSO4, PSA, C_18_, and GCB as dispersive solid-phase extraction sorbent. Figure S5: Peak intensity of different concentrations of deltamethrin at 559 cm^−1^ (A) and 999 cm^−1^ (B). Table S1: The assignment of Raman peaks of deltamethrin, Table S2. The assignment of *Corydalis* extract. Table S3: Detection limit of PLSR models for deltamethrin in *Corydalis* title. All authors have read and agreed to the published version of the manuscript.
