# Supplementary material for: Rapid Quantitative Detection of Deltamethrin in Corydalis yanhusuo by SERS Coupled with Multi-Walled Carbon Nanotubes
Source: Molecules. 2020 Sep 7;25(18):4081. doi: 10.3390/molecules25184081 (PMC7570915; doi:10.3390/molecules25184081)
Supplement: Supplementary file 1 [file molecules-25-04081-s001.pdf]

Supplementary information

Rapid Quantitative Detection of Deltamethrin in *Corydalis yanhusuo* by SERS Coupled with Multi-walled Carbon Nanotubes

Hui Zhang [1,2], Pengcheng Nie [1,2,3], Zhengyan Xia [4,*], Xuping Feng [1,2], Xiaoxi Liu [1,2] and Yong He [1,2]

1 College of Biosystems Engineering and Food Science, Zhejiang University, Hangzhou 310058, China; 21813051@zju.edu.cn (H.Z.); npc2012@zju.edu.cn (P.N.); pimmmx@163.com (X.F.); 21813015@zju.edu.cn (X.L.); zjuheyong@sina.com (Y.H.)

2 Key Laboratory of Spectroscopy Sensing, Ministry of Agriculture, P. R, Hangzhou 310058, China.

3 West Electronic Business Company Limited, Yinchuan 750000, China.

4 School of Medcine, Zhejiang University City College, Hangzhou 310015, China.

* Correspondence:xiazy@zucc.edu.cn; Tel.: +xx-xxxx-xxx-xxxx

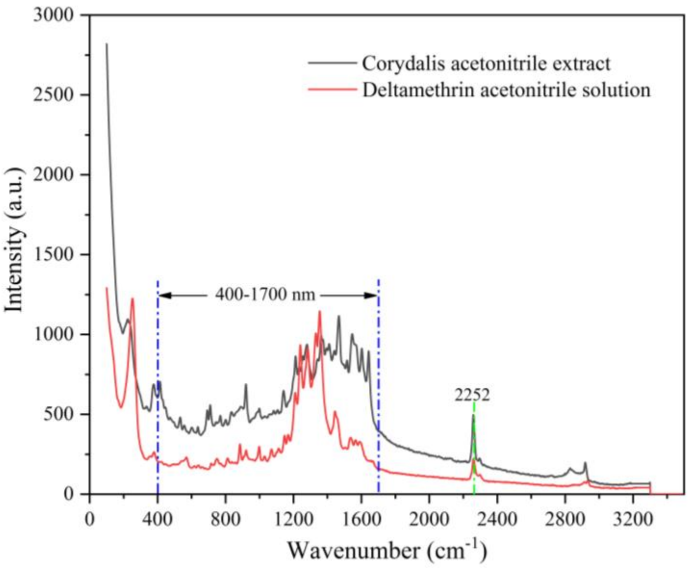

Figure S1. SERS spectra of deltamethrin solution and corydalis extract.

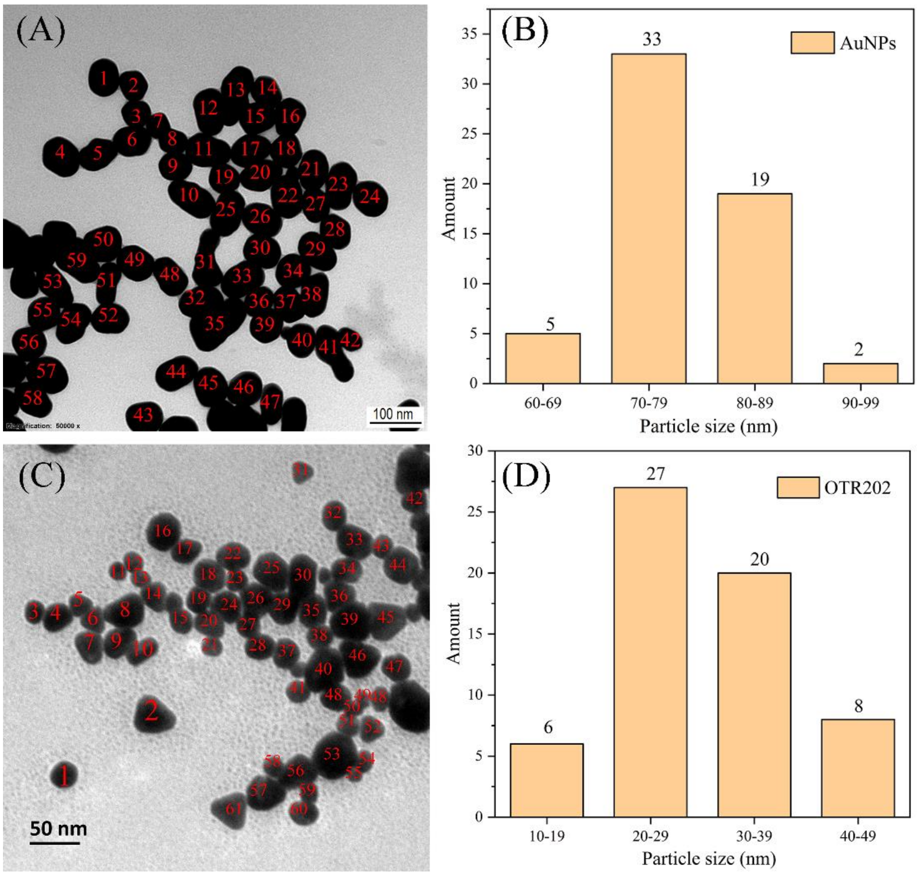

Figure S2. Size distribution of nanoparticles. (**A**): particle marking for size statistics of AuNPs; (**B**): size distribution of AuNPs; (**C**): particle marking for size statistics of OTR202; (**D**): size distribution of OTR202.

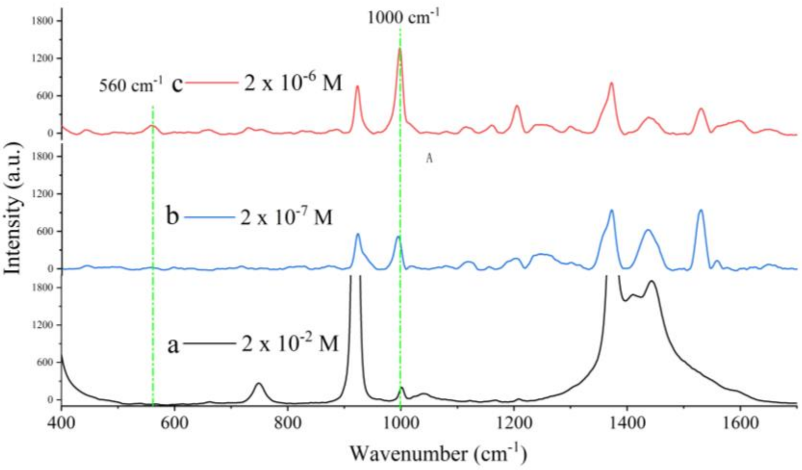

Figure S3. Raman spectroscopy of deltamethrin. (**a**) The normal Raman spectra of 2 × 10^{-2} M deltamethrin. SERS spectra of deltamethrin concentrations of 2 × 10^{-7} M (b) and 2 × 10^{-6} M (**c**).

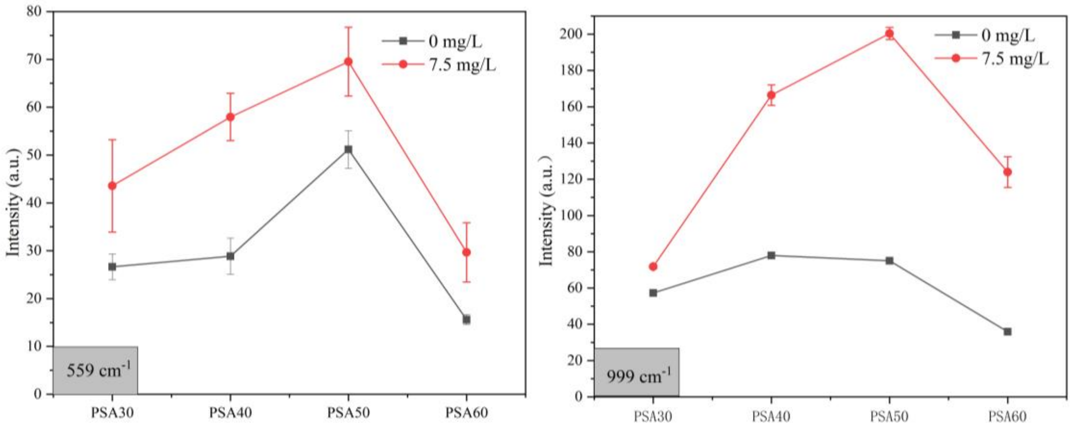

Figure S4. Peak intensity at 999cm^{-1} and 559cm^{-1} with MgSO4, PSA, C18, and GCB as dispersive solid-phase extraction sorbent.

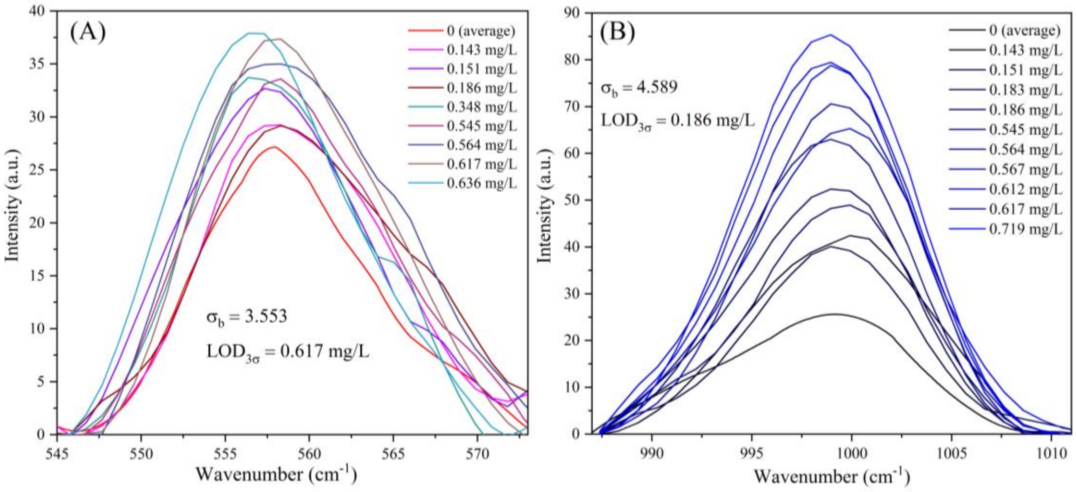

Figure S5. Peak intensity of different concentrations of deltamethrin at 559cm^{-1} (A) and 999cm^{-1} (B).

Table S1. The assignment of Raman peaks of deltamethrin.

DFT (cm^{-1})	Solid (cm^{-1})	SERS (cm^{-1})	Assignments
414	408(w)	-	υ_{ring}
553	554(m)	565(m)	υ(C-Br)ip + υ(C-C)
658	643(w)	655(w)	υ_{ring}+υ(C-C-C)
736	735(w)	748(w)	δ(C-H)
776	776(w)	777(s)	υ_{ring}+δ(C=N)
823	821(w)	810(w)	$\upsilon_{breathe}$
881	870(w)	883(m)	δ(C-H)
906	922(m)	920(m)	υ_{ring+}δ(C-H)
-	964(m)	960(w)	δ(C-H)
987	1000(vs)	999(vs)	υ_{ring+}δ(C-C)
1027	1024(m)	1021(w)	υ_{ring}
-	1046(w)	1048(w)	υ(C-C)
1065	1069(s)	1069(m)	δ(C-H)
1117	1116(w)	1114(w)	δ(C-H)
1154	1166(w)	1165(w)	υ_{ring}+δ(C-H)
1191	1206(m)	1207(s)	υ(C-C)
1248	1250(w)	1241(s)	υ_{ring}+δ(C-H)
1278	1280(w)	1285(m)	δ(C-H)
1328	1326(w)	1330(m)	δ(C-H)
1410	1403(w)	1405(w)	δ(C-H)
1440	1446(w)	1446(s)	δ(C-H)
1470	1463(w)	-	δ(C-H)
1600	1593(s)	1570(m)	υ(C=C)
1613	1610(m)	1602(m)	υ(C=C)
1721	1733(w)	1741(w)	υ(C=O)

Note: vs = very strong; s = strong; m = medium; w = weak; υ = stretching; δ = deformable vibration.

Table S2. The assignment of Corydalis extract.

SERS (cm^{-1})	Assignments	Reference
414(m)	υ_{ring}	Table S1
448(w)	δ(C-C-C)	[1]
532(m)	δ(C-C-C-C)	[1]
603(w)	δ(C-C-C) +υ(C-C) + δ(O-C-N)	[1]
690(w)	Ring breath	[2]
710(m)	$\upsilon_{breathe}$	Table S1
772(m)	υ_{ring}+δ(C=N)	Table S1
798(w)	δ(H-C-C-H)	[3]
833(w)	υ(C-C)	[1]
1140(m)	δ(H-C-C)	[1]
1212(s)	υ(N-C) +δ(C-H)	[4]
1234(w)	υ(C-C)	[1]
1253(w)	υ(C–N)	[5]
1282(vs)	δ(C-H)	Table S1
1339(w)	δ(H-C-C) + υ(N-C)	[1]
1363(s)	δ(C-H)	[6]
1412(w)	υ(C=C) + δ(H-C-C)	[1]
1468(vs)	δ(C-H)	[7]
1515(m)	υ(C=C)	[8]
1545(s)	υ(C=-C) + δ(C-H)	[7]
1570(s)	υ(C=C)	[5]
1604(s)	υ(C=C)	Table S1
1643(vs)	υ(C-C)	[9]

Note: vs = very strong; s = strong; m = medium; w = weak; υ = stretching; δ = deformable vibration.

Table 3. Detection limit of PLSR models for deltamethrin in Corydalis.

Spectral Band (cm^{-1})	Standard Error of Intercept (mg/L)	Slope	Detection Limit (mg/L)
545–573	0.220	0.989	0.667
987–1011	0.234	1.051	0.668
545–573+987–1011	0.176	1.090	0.484

References

1. Unsalan, O.; Sert, Y.; Ari, H.; Simão, A.; Yilmaz, A.; Böyükata, M.; Bolukbasi, O.; Bolelli, K.; Yalcin, I. Micro-Raman, Mid-IR, Far-IR and DFT studies on 2-[4-(4-Fluorobenzamido)phenyl]benzothiazole. *Spectrochim. Acta Part A: Mol. Biomol. Spectrosc.* **2014**, *125*, 414–421, doi:10.1016/j.saa.2014.01.118.
2. Chen, Q.; Jiao, T.; Yang, M.; Li, H.; Ahmad, W.; Hassan, M.; Guo, Z.; Ali, S. Pre etched Ag nanocluster as SERS substrate for the rapid quantification of AFB1 in peanut oil via DFT coupled multivariate calibration. *Spectrochim. Acta Part A: Mol. Biomol. Spectrosc.* **2020**, *239*, 118411, doi:10.1016/j.saa.2020.118411.
3. Unsalan, O.; Arı, H.; Altunayar-Unsalan, C.; Bolelli, K.; Boyukata, M.; Yalcin, I. FTIR, Raman and DFT studies on 2-[4-(4-ethylbenzamido)phenyl]benzothiazole and 2-[4-(4-nitrobenzamido)phenyl]benzothiazole supported by differential scanning calorimetry. *J. Mol. Struct.* **2020**, *1218*, 128454, doi:10.1016/j.molstruc.2020.128454.
4. Cañamares, M.V.; Pozzi, F.; Lombardi, J.R. Raman, SERS, and DFT Analysis of the Main Alkaloids Contained in Syrian Rue. *J. Phys. Chem. C* **2019**, *123*, 9262–9271, doi:10.1021/acs.jpcc.9b01355.
5. Liu, B.; Zhou, P.; Liu, X.; Sun, X.; Li, H.; Lin, M. Detection of Pesticides in Fruits by Surface-Enhanced Raman Spectroscopy Coupled with Gold Nanostructures. *Food Bioprocess Technol.* **2012**, *6*, 710–718, doi:10.1007/s11947-011-0774-5.
6. Oliveira, R.P.; Demuner, A.J.; De Alvarenga, E.S.; Parma, M.C.; Barbosa, L.C.A.; Guimarães, L.D.M.; Aguiar, A.R. Experimental and theoretical studies on the characterization of monocrotaline by infrared and Raman spectroscopies. *J. Mol. Struct.* **2017**, *1135*, 228–233, doi:10.1016/j.molstruc.2017.01.050.
7. Cañamares, M.V.; Lombardi, J.R.; Leona, M. Surface-enhanced Raman scattering of protoberberine alkaloids. *J. Raman Spectrosc.* **2008**, *39*, 1907–1914, doi:10.1002/jrs.2057.
8. Frosch, T.; Schmitt, M.; Schenzel, K.; Faber, J.H.; Bringmann, G.; Kiefer, W.; Popp, J. In vivo localization and identification of the antiplasmodial alkaloid dioncophylline A in the tropical lianaTriphyophyllum peltatum by a combination of fluorescence, near infrared Fourier transform Raman microscopy, and density functional theory calculations. *Biopolym.* **2006**, *82*, 295–300, doi:10.1002/bip.20459.
9. Singh, S.; Singh, H.; Karthick, T.; Tandon, P.; Dethe, D.H.; Erande, R.D. Conformational Study and Vibrational Spectroscopic (FT-IR and FT-Raman) Analysis of an Alkaloid–Borreverine Derivative. *Anal. Sci.* **2017**, *33*, 99–104, doi:10.2116/analsci.33.99.
